# “The Power of a Firm’s Benevolent Act”: The Influence of Work Overload on Turnover Intention, the Mediating Role of Meaningfulness of Work and the Moderating Effect of CSR Activities

**DOI:** 10.3390/ijerph18073780

**Published:** 2021-04-05

**Authors:** Yun-Seok Hwang, Byung-Jik Kim

**Affiliations:** 1 Department of HRD Consulting, University of Ulsan, Ulsan 44610, Korea; yoon7749@hrdkorea.or.kr; 2 College of Business Administration, University of Ulsan, Ulsan 44610, Korea

**Keywords:** work overload, meaningfulness of work, turnover intention, corporate social responsibility, moderated mediation model

## Abstract

Although previous works have examined how work overload affects the perceptions, attitudes, and behaviors of members in an organization, those studies have paid insufficient attention to the mediating and contingent factors in the work overload–turnover intention link from the perspective of positive psychology. Considering the theoretical and practical value and importance of positive psychology, studies that use it to investigate underlying processes are needed. Also, existing studies on work overload have focused on the moderating role of individual-level variables to reduce the negative effects of work overload, mostly ignoring the importance of organizational-level moderators. To resolve those issues, we hypothesize that the meaningfulness of an employee’s work mediates the relationship between work overload and turnover intention. Corporate social responsibility (CSR) practices could moderate the association between work overload and the meaningfulness of work. Using a three-wave data set gathered from 356 currently working employees in South Korea, we reveal not only that the meaningfulness of work mediates the work overload–turnover intention link, but also that CSR activities play a buffering role in the work overload–meaningfulness of work link. Our findings suggest that, from the perspective of positive psychology, the degree of meaningfulness of work (as a mediator) and CSR activities (as a moderator) function as intermediating mechanisms in the work overload–turnover intention link.

## 1. Introduction

Due to the extremely competitive business environment, employees in almost all kinds of firms are suffering from a considerable amount of work. They experience increased mental/physical burden and pain that are caused by excessive duties at work. This phenomenon is called work overload, the degree to which an employee perceives that the amount of work or its intensity is excessive when performing a job [1]. Previous studies have reported that work overload critically influences the perceptions, attitudes, and behavior of employees. For example, work overload is known to reduce the level of employee job satisfaction, organizational commitment, organizational identification, satisfaction at work, self-esteem, confidence, and motivation, and it increases employee job stress, work–family conflict, emotional exhaustion, burnout, psychological pressure, and turnover intention [2,3,4,5,6,7,8,9,10,11,12,13].

Although some studies have examined how work overload affects turnover intention, including its underlying mechanisms, some issues have not yet been sufficiently explained. First, previous studies have left important positive psychology-related constructs such as the meaningfulness of work, gratitude, forgiveness, and prosocial motivation relatively underexplored in explaining the mediating mechanisms between work overload and various organizational outcomes [14]. Positive psychology examines various organizational phenomena by scrutinizing positive processes, attributes, and outcomes rather than negative aspects [14]. For example, many previous studies have investigated the effects of work overload on negative variables such as emotional exhaustion, job tension, and job stress [4,11,15]. However, considering the theoretical and practical value and importance of positive psychology [14], studies that investigate underlying intermediating processes from that perspective are needed. Second, the existing literature on work overload has focused on the moderating role of individual-level variables in reducing the negative effects of work overload. For example, Jex and colleagues [8] reported that the negative effects of work overload on job stress were reduced when employees felt that their organization cared for them and supported them. Other studies have concentrated on other individual-level factors that reduce the negative effects of work overload, including employee coping strategies [13], work experience [16], political techniques [11], social support of peers [3], and job diversity [17]. In other words, the collective- or organizational-level moderating factors that could decrease the harmful effects of work overload have been left underexplored. Although research that examines individual-level contingent factors is meaningful, individuals are systemically embedded in organizations, so it is necessary to investigate organizational- and macro-level moderating variables when explaining the influence of work overload. Third, from a methodological point of view, existing studies have generally used cross-sectional datasets, which suffer from common method bias. Therefore, a longitudinal approach is needed.

To address those issues, we suggest that work overload could increase the degree of an employee’s turnover intention via the mediating effect of the meaningfulness of work. The meaningfulness of work is defined as the general beliefs, values, and attitudes that members of an organization have about their work [18] and as the degree to which members of an organization consider their work to be valuable and important [19]. The meaningfulness of work is thus closely related to other positive attitudes and behaviors in an organization, such as organizational identification, organizational commitment, job satisfaction, work engagement, intrinsic motivation, turnover intention, organizational citizenship behavior, and in-role/extra-role performance [20,21,22,23]. Employees who feel a sense of work overload might feel that their work does not have enough meaning because they are experiencing an increased level of job stress, job strain, and burnout, along with a decreased level of self-efficacy and self-esteem [2,7,11,12,13,20]. We suggest that the meaningfulness of work could decrease the level of an employee’s turnover intention, which is the degree to which a member wants to leave his or her current job or organization to seek another one [24]. When employees experience a high level of meaningfulness in their work, their turnover intention could be reduced by a sense of felt obligation to their organization driven by the feelings of fullness they experience while they are part of it [25,26].

As explained above, the argument that work overload negatively affects the meaningfulness of work is reasonable and acceptable. However, the theoretical association between the variables might not be valid in all situations because various contingent or contextual variables might affect that relationship. Among the various potential moderating factors, based on the person-organization fit (PO fit) framework [27,28], we suggest that corporate social responsibility (CSR), as a macro- and organizational-level variable, might function as a moderating variable that mitigates the negative effects of work overload on an employee’s meaningfulness of work. CSR describes the efforts of firms to enhance the welfare of various stakeholders, such as shareholders, employees, customers, local communities, and the environment, while operating a business [29,30,31]. CSR may function as a critical organizational context that interacts with an employee’s sense of work overload, eventually moderating the influence of work overload on turnover intention.

For example, when an organization actively performs CSR activities, even if the members feel a sense of work overload, the negative impacts of work overload may be reduced. This is because when an organization fulfills its social responsibilities, members are likely to feel pride, organizational identification, organizational commitment, meaningfulness of work, and job satisfaction [27,28,29,30]. Those positive emotions can offset the negative effects of work overload. On the other hand, if an organization rarely practices CSR, members experiencing work overload might not feel proud or satisfied with it and might not feel that their work is meaningful, leaving them vulnerable to the negative effects of work overload.

In this study, we investigate the effect of work overload on turnover intention through the mediating role of meaningfulness of work. Also, we suggest that CSR activities function as a contingent factor that moderates the relationship between work overload and meaningfulness of work. Our study makes the following contributions. First, by emphasizing the mediating role of meaningfulness of work, we explore the underlying mechanism of the work overload–turnover intention link from the perspective of positive psychology. Second, we reveal that CSR practices, an organizational level variable, moderates the negative influence of work overload. Third, from a methodological point of view, we bypass the limitations of existing studies based on a cross-sectional research design by taking a longitudinal (i.e., 3-wave time-lagged) approach.

## 2. Theory and Hypotheses

### 2.1. Work Overload and Meaningfulness of Work

We suggest that work overload could decrease the level of meaningfulness of work for employees. Work overload is a sub-dimensional concept of job demand [1,2,16], which is the degree to which employees perceive that the amount of work or its intensity are excessive [1]. Previous studies have reported that work overload critically influences the perceptions, attitudes, and behavior of employees. For example, work overload is known to reduce job satisfaction or satisfaction at work [6,9], and increase employee turnover intentions [8].

By applying a psychological perspective, prior studies on the meaningfulness of work have assumed that it is rooted in the subjective interpretation of each member’s work experiences and interactions within an organization [19,32,33]. As experience at work becomes an increasingly important area of life [34], and as employees expect their work to satisfy more psychological, social, and economic needs [35], the academic importance of the meaningfulness of work is gradually increasing.

Previous studies on the meaningfulness of work have reported that self-efficacy, competence, and self-esteem are the primary antecedent factors that influence it [20,36,37,38,39,40]. For example, when members feel that they can make positive changes by effectively influencing the organization and their colleagues, i.e., they have self-efficacy, they are likely to experience a high level of meaningfulness of work [20,40,41]. In addition, those members are likely to grow by successfully problem-solving to complete their assigned tasks, which could enhance their self-competence and pride, positively contributing to their sense that their work is meaningful [37,42,43].

Therefore, by directly decreasing the level of an employee’s self-efficacy, self-esteem, and competence, work overload deteriorates the meaningfulness of their work. In a state of work overload, members can feel great psychological stress, tension, and exhaustion, which can reduce their levels of self-esteem, self-confidence, and motivation [7,11,12,13]. Those negative psychological states substantially decrease the meaningfulness of work. Therefore, we propose the following hypothesis.

**Hypothesis 1** **(H1).** *Work overload will have a negative (-) effect on the meaningfulness of work.*

### 2.2. The Meaningfulness of Work and Turnover Intention

Members generally want their work to be more than just a means of making money or spending time, so they try to find meaning in what they do [44]. The meaning that members experience within an organization greatly influences how they interpret events that occur in the workplace, what experiences they have in the organization, and how they perform their duties [20,40]. Previous studies have reported that the meaningfulness of work is closely and positively related to employee job satisfaction, organizational commitment, and intrinsic motivation [20,23,45,46,47]. Those positive perceptions, in turn, increase the positive emotions and psychological states of organizational members [48,49].

One fundamental reason that members experience their work as meaningful lies in the organization. Members who experience their work as meaningful are likely to recognize that the organization for which they do that work has provided them with positive experiences. Put another way, members whose work feels meaningful to them perceive that they receive significant support from their organization. According to social exchange theory, enhanced perceived organizational support facilitates a sense of felt obligation to repay the organization for its support [25,26,50], which also satisfies members’ socio-psychological needs [51]. Those inner states and experiences increase an employee’s intention to remain part of the organization. In contrast, if the members of an organization do not find their work for that organization to be meaningful, they do not have positive experiences such as perceived organization support, job satisfaction, organizational commitment, and intrinsic motivation. When those employees experience work overload, they often struggle to find a reason to remain in the organization. Therefore, we make the following hypothesis.

**Hypothesis 2** **(H2).** *The meaningfulness of work will have a negative (-) effect on turnover intention.*

### 2.3. Mediating Effect of Meaningfulness of Work in an Association between Work Overload and Turnover Intention

By integrating the relationships among our research variables (work overload, meaningfulness of work, and turnover intention), we suggest that work overload will increase employee turnover intention via the mediating effect of the meaningfulness of work. From the perspective of positive psychology, the positivity of the meaningfulness of work is likely to be decreased by the perception of work overload. Therefore, we propose the following hypothesis.

**Hypothesis 3** **(H3).** *The meaningfulness of work will mediate the relationship between work overload and turnover intention.*

### 2.4. Moderating Role of CSR in the Work Overload–Meaningfulness of Work Link

As mentioned above, the relationship between work overload and meaningfulness of work has been theoretically and empirically validated by previous works. However, it seems too simple and naive to believe that work overload will always decrease the level of meaningfulness of work in all situations and conditions as members tend to react differently to the work overload. Although we acknowledge that work overload will decrease the perceived meaningfulness of work, we also suggest that several contingent and contextual variables could moderate that association. Among those potential moderators, based on the person-organization fit (PO fit) perspective [27,28], we propose that CSR, as a macro- and organizational-level variable, is likely to function as a contingent factor that mitigates the negative influence of work overload on meaningfulness of work.

PO fit perspective has played an important role in explaining the employee’s perceptions, attitudes, and behaviors interaction in an organization [27,28]. PO fit has been defined as ‘‘the compatibility between people and organization that occurs when at least one entity provides what the other needs, or both share similar fundamental characteristics” [28] (p. 4–5). Employees who fit well with their organization tend to have more positive attitudes and behaviors such as job satisfaction, organizational commitment, turnover intention, organizational identification, and citizenship behavior [27,28]. According to the PO fit framework, CSR, as an important organizational practice or system, can play a critical role as an organizational context that interacts with individual employee’s perceptions such as a sense of work overload [27]. Then, it would eventually moderate the influence of work overload on turnover intention.

CSR has been shown to not only improve organizational performance by functioning as an effective corporate strategy [52,53], but also positively affect organizational identification, organizational commitment, job satisfaction, and organizational citizenship behavior [27,54,55,56,57,58]. Based on those positive effects of CSR activities, we suggest that CSR practices could play a moderating role in the relationship between work overload and the meaningfulness of work.

For instance, when the level of CSR activities is high, the negative influences of work overload are likely to be decreased, even if the employees feel a sense of psychological burden due to the work overload. This is since when an organization actively performs its social responsibilities, employees would feel a sense of pride, organizational identification, organizational commitment, and job satisfaction. Then, the positive emotions and psychological states of employees are likely to mitigate the negative impacts of work overload. In contrast, when an organization rarely conducts its social responsibility activities, employees who are suffering from psychological stress and burden due to the excessive task would not be proud of their organization, are not be satisfied with it, and do not perceive the meaningfulness of work. Eventually, the negative influences of work overload may not be adequately resolved.

**Hypothesis 4** **(H4).** *CSR would moderate the relationship between work overload and meaningfulness of work.*

**Figure 1:**
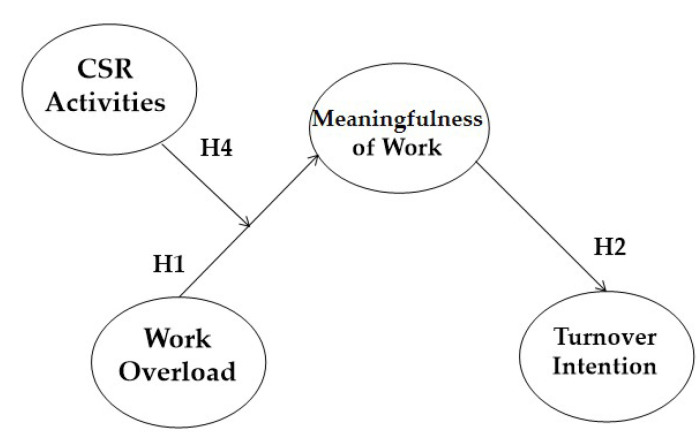
Theoretical Model.

Taken together, we propose that work overload increases turnover intention through a mediating effect of meaningfulness of work. Moreover, CSR activities would moderate the work overload-meaningfulness of work link (Please See Figure 1). 

## 3. Method

### 3.1. Data Gathering

Using an online survey system, we collected data from currently working Korean employees at three different time points. The surveys were conducted by a large online research firm in South Korea with about 1,300,000 panelists. The research firm randomly selected respondents to reduce the possibility of sampling bias in employee characteristics that could influence the results of our research (e.g., gender, tenure, position, and education). The online system’s operating functions allowed us to track who responded to our survey, confirming that participants from time point 1 to time point 3 were the same. We believe that this paper may reduce the harmful influence of common method bias (CMB) by measuring each research variable from different time point.

At time 1770 participants responded to our initial survey. At time 2550 employees participated in our second survey, and at time 3370 employees responded to our third and final survey. The interval between surveys was four or five weeks. Our survey system was open for two or three days each at each time point to provide enough time for participants to respond. When the system was open, participants could access it whenever they wanted. After we collected the data, we eliminated surveys with missing data. Ultimately, we used data from 356 employees who provided complete answers to all three surveys in our analysis. The characteristics of the participants are shown in Table 1.

### 3.2. Measures

Because the original items were developed in English, we translated them into Korean. Then, bilingual researchers back-translated the items. All research variables were measured using a five-point Likert scale (1 = strongly disagree to 5 = strongly agree). We calculated the internal consistency of all variables using Cronbach’s alpha coefficients (Appendix A).

#### 3.2.1. Work Overload (Time Point One, Gathered from Employees)

To measure the degree of employee work overload, we used 5 items from previously published scales [59,60]. All items included in our study are (a) “I am pressured to work long hours”; (b) “I have unachievable deadlines”; (c) “I have to work very fast”; (d) “I have to work very intensively”; and (e) “I have unrealistic time pressures”. The Cronbach alpha value in this study was = 0.92.

#### 3.2.2. Meaningfulness of Work (Time Point 2, Collected from Employees)

To measure the degree to which employees found meaningfulness in their work, we used 5 items from previous studies [22,23]. Typical items included in our study were: (a) “The work that I do is meaningful”; (b) “The work that I do makes the world a better place”; and (c) “My work is one of the most important things in my life.” The Cronbach alpha value in this study was = 0.88.

#### 3.2.3. Turnover Intention of Employees (Time Point 3, Collected from Employees)

We measured employees’ turnover intention using three items from a scale developed by Konovsky and Cropanzano [61]. The items were (a) “How likely is it that you will look for a job outside of this organization during the next year?”, (b) “How often do you think about quitting your job at this organization?”, and (c) “If it were possible, how much would you like to get a new job?”. The Cronbach alpha value in this study was = 0.88.

#### 3.2.4. Corporate Social Responsibility (Time Point One, Gathered from Employees)

We measured the level of CSR in each organization by adapting 12 items from the work of Farooq and colleagues [62], which they took from Turker’s original CSR scale [63]. Although Turker’s original scale combined the environmental and community dimensions of CSR, the empirical work of Farooq and colleagues split the two. Thus, our scale consists of four domains: environment, community, employee, and customer. Each of the four dimensions contains three items and represents corresponding stakeholders in social responsibility. For the environmental dimension, the items are: “our company participates in activities which aim to protect and improve the quality of the natural environment”; “our company implements special programs to minimize its negative impact on the natural environment”; and “our company targets sustainable growth which considers future generations.” For the community dimension, the items are: “our company contributes to campaigns and projects that promote the well-being of society”; “our company emphasizes the importance of its social responsibilities to society”; and “our company actively participates in voluntarily donations to charities and nongovernmental organizations.” For the employee dimension, the items are: “management at our company is primarily concerned with employees’ needs and wants”; “our company policies encourage employees to develop their skills and careers”; and “our company supports employees’ growth and development.” For the customer dimension, the items are: “our company respects consumer rights beyond legal requirements”; “our company provides full and accurate information about its products to its customers”; and “customer satisfaction is highly important for our company.” Those items have been used in previous studies conducted in South Korean contexts [64,65,66]. Because we were interested in the interaction effect between work overload and CSR, we collected responses to these items only at time 1. The reliability coefficient in this study was 0.90.

#### 3.2.5. Control Variables

Turnover intention was controlled by employee’s tenure (in months), gender, position, and educational level. To maintain the consistency of this study, this paper collected the control variables at Time two.

### 3.3. Analytical Strategy

The associations among the variables were evaluated by a Pearson correlation analysis (*n* = 356). As suggested by Anderson and Gerbing [67], a two-step approach that consists of the measurement and the structural model were applied. For checking the validity of the measurement model, we performed a CFA (confirmatory factor analysis) for our research variables. Then we performed a structural equation modeling (SEM) analysis by building a moderated mediation model to test our structural model. We used the maximum likelihood (ML) estimator to perform the SEM. Moreover, to evaluate our mediation hypothesis, we performed a bootstrapping procedure by using the 95% bias-corrected confidence interval (CI) to evaluate the mean indirect mediation. If the CI does exclude 0, it is interpreted that the indirect effect was statistically significant with a 0.05 level.

To test if the model fit were adequate, we used a variety of goodness-of-fit indices including the comparative fit index (CFI), the Tucker–Lewis index (TLI), and the root mean square error of approximation (RMSEA). Extant studies reported that an adequate fit is indicated by CFI and TLI values bigger than 0.90 and an RMSEA value of smaller than or equal to 0.06 [68]. Lastly, a bootstrapping analysis was performed to check if the indirect effect were significant [69].

## 4. Results

### 4.1. Descriptive Statistics

The result of correlation analysis is described in Table 2. The study variables including work overload, CSR activities, meaningfulness of work, and turnover intention were significantly associated.

### 4.2. Measurement Model

We performed confirmatory factor analyses (CFAs) for all 25 items to examine the goodness-of-fit of the measurement model. Because four psychometric constructs (work overload, meaningfulness of work, CSR activities, and turnover intention) are incorporated in our research model, we identified their discriminant validity. Our hypothesized 4-factor model showed a good fit (χ^2^ (df = 110) = 194.231; CFI = 0.976; TLI = 0.971; RMSEA = 0.046). Then, we performed a series of chi-square difference tests by consequently comparing the 4-factor model to a 3-factor (χ^2^ (df = 113) = 806.976; CFI = 0.804; TLI = 0.764; RMSEA = 0.132), 2-factor (χ^2^ (df = 115) = 1041.052; CFI = 0.739; TLI = 0.691; RMSEA = 0.151), and one-factor (χ^2^ (df = 116) = 1592.355; CFI = 0.584; TLI = 0.512; RMSEA = 0.189) models. The results of the chi-square difference tests indicated that the 4-factor one was best. Therefore, we suggest that the four variables have an adequate level of discriminant validity.

### 4.3. Structural Model

In this research, we established a moderated mediation model that includes both mediating and moderating structures between work overload and employee turnover intentions. In the mediating structure, the work overload–turnover intention link was mediated by the meaningfulness of work. In the moderating structure, CSR moderates the effect of work overload on the meaningfulness of work.

To identify if there is a multi-collinearity bias between our independent variables (i.e., work overload and CSR), this paper checked the variance inflation factors (VIF) and tolerances [70]. The VIF values for work overload and CSR are 1.01 and 1.01. In addition, the tolerance values are 0.99 and 0.99. Because the VIF scores are smaller than 10 as well as the tolerance scores above 0.2, this paper suggests that work overload and CSR are relatively free from the problem of multi-collinearity.

#### 4.3.1. Results of Mediation Analysis

**Figure 2:**
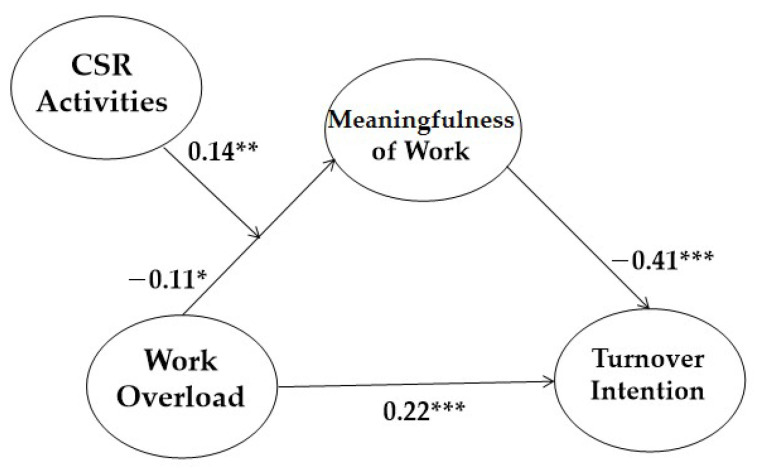
The Result of Coefficient Values of our Research Model.

To find the best model, we conducted SEM analyses and a chi-square difference test with alternative models, including the full mediation model and a partial mediation model (Figure 2). The partial mediation model includes a direct path from work overload to turnover intention. The fit indices of all the alternative models were adequate. The results of the chi-square difference tests suggest that the partial mediation model (χ^2^ (df = 136) = 229.866; CFI = 0.971; TLI = 0.963; RMSEA = 0.044) has a better fit than the full mediation model (χ^2^ (df = 137) = 246.322; CFI = 0.966; TLI = 0.957; RMSEA = 0.047), indicating that work overload affects the level of turnover intention, both directly and indirectly.

**Figure 3:**
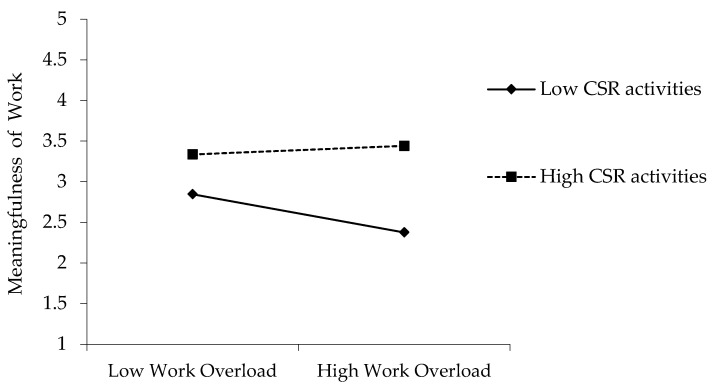
Moderating Effect of CSR in the Work Overload–Meaningfulness of Work link.

The control variables (gender, position, tenure, and education level) were statistically non-significant except for gender (β = 0.12, *p* < 0.05), and tenure (β = −0.144, *p* < 0.01). Incorporating the control variables, our model shows supporting result for all hypotheses. Work overload decreases the level of meaningfulness of work (β = −0.112, *p* < 0.05), supporting Hypothesis 1, and the meaningfulness of work diminishes turnover intention (β = −0.405, *p* < 0.001), supporting Hypothesis 2 (Figure 3).

#### 4.3.2. Bootstrapping

Bootstrapping analyses were conducted by using a sample of 10,000 [69] to test Hypothesis 3. Indirect mediation effects are significant at the 5% level if the 95% bias-corrected confidence interval (CI) for the mean indirect mediation effects does not include zero [69]. Our results show that the bias-corrected CI for the mean indirect effects on the paths did not include zero (95% CI = [0.005, 0.094]). Thus, we can conclude that Hypotheses 3 is supported. The direct, indirect, and total effects of the paths from work overload to turnover intention are provided in Table 3.

#### 4.3.3. Results of Moderation Analysis

The moderating influence of CSR activities on the relationship between work overload and the meaningfulness of work was evaluated using the moderated mediation model. To make an interaction term, we conducted a mean-centering procedure. Centered variables not only estimate interaction terms in an efficient way, they also decrease multicollinearity among the variables [70].

The coefficient value of the interaction term was statistically significant (β = 0.139, *p* < 0.01), indicating that CSR positively moderates the work overload–meaningfulness of work link. Thus, a high degree of CSR practices buffers the negative influence that work overload has on the meaningfulness of work, supporting Hypothesis 4.

## 5. Discussion

Using three waves of time-lagged data, we have shown that the perceived meaningfulness of work plays a mediating role in the relationship between work overload and turnover intention. We have also revealed that CSR activities function as a buffering factor in the link between work overload and the meaningfulness of work. This research contributes to the literature on work overload, meaningfulness of work, turnover intention, and CSR by revealing a mediator (meaningfulness of work) and a moderator (CSR practices) that describe why and when work overload influences turnover intention.

### 5.1. Theoretical Implications

The theoretical implications of this study are as follows. First, we have interpreted work overload from the perspective of positive psychology [14], emphasizing the importance of variables such as meaningfulness of work and social responsibility and revealing that work overload not only lowers employee positivity (i.e., meaningfulness of work) but also ultimately increases turnover intention. To be specific, we have demonstrated that an employee’s meaningfulness of work, as a representative variable of positive psychology, functions as a mediating mechanism in explaining the influence of work overload on turnover intention.

Second, we have shown that CSR activities play an important contingent role in the relationship between work overload and meaningfulness of work. From an employee’s point of view, working for an organization that is recognized as carrying out a lot of social activities mitigates the negative effects of work overload. This indicates the importance of job analysis, which measures the degree of work overload within an organization, as well as the necessity of CSR practices.

### 5.2. Practical Implications

The results of this study provide practical implications for corporate managers who want to reduce the negative influence of work overload. First, by understanding an underlying process in the relationship between work overload and turnover intention, top management teams or leaders can estimate how severe the negative effects of work overload are. This research demonstrates that work overload can increase the level of employee turnover intention by decreasing the meaningfulness of their work. Thus, if top management teams or leaders want to understand the effects of work overload, they must carefully monitor both the levels and changes in employee attitudes (i.e., meaningfulness of work).

Second, based on our finding that CSR practices function as a buffering factor that diminishes the negative effects of work overload, CSR can be regarded as an effective investment rather than merely a moral duty. Given that human resources are a critical factor that provides firms with a significant competitive advantage [71], decreasing the degree of employee turnover intention through CSR activities could be as a reasonable strategy for companies. Therefore, leaders should not only try to communicate and interact with their employees about the CSR practices, but also supply adequate rewards for the employees to maximize active participation in CSR activities. By regularly communicating about the organizations’ socially responsible practices, the top management team can establish a CSR-oriented culture in the organization [72].

### 5.3. Limitations and Suggestions for Future Studies

Despite its theoretical and practical implications, our study has several limitations. First, this research could not resolve the issue of potential alternative explanations for mediating effects. To be specific, we did not consider competitor-variables to meaningfulness of work in explaining the work overload–turnover intention link. Our finding that the direct path from work overload to turnover intention is significant suggests that other mediators might participate in that association. For example, stress-related variables such as job stress or burnout could function as a more direct mediator to explain the relationship.

Second, we used data only from employees in South Korean companies. Considering that employee perceptions and attitudes are significantly influenced by their cultural background, we must be cautious in interpreting and applying our results to employees in other cultures. To be specific, cultural differences between Eastern and Western societies are likely to influence how employees perceive CSR practices of the firm. Western cultures tend to emphasize the importance of socially prescribed obligations (e.g., CSR activities); therefore, the members may be more sensitive to the social duties [73,74]. Since this paper gathered the data from employees of South Korean companies, cautious interpretation of the results is necessary when the findings are interpreted in the context of different cultures [73,75]. Despite the universality of the spirit of CSR [74,76], Korean employees are likely to respond differently to the request for the social responsibilities than in Western cultures. Therefore, further works should consider this.

Third, we could not utilize objective scales to measure CSR activities. Although extant research on CSR has proposed that subjective measures such as employees’ perceptions on CSR would more adequately reflect the real phenomena about CSR than objective CSR scales [77], this paper recommend future research to utilize both subjective and objective CSR measures.

Fourth, our research cannot be free from common method bias because work overload, meaningfulness of work, the degree of CSR activities, and employee levels of turnover intention were all reported by the same people. Although our dataset has a time-lagged structure, this fundamental limitation should be acknowledged. Although the results of our CFA demonstrate that the variables in our research model are distinctive, future studies should deal with this issue.

Fifth, this research could not consider the important role of the firm’ pre-established reputation or firm status in determining employee’s turnover intention. Given that the characteristics of a firm would critically influence employee’s perceptions, attitudes, and behaviors in an organization, future studies should deal with this issue.

Lastly, this paper does not apply multi-level theories and perspectives. The structure of our dataset could not be multi-leveled. Considering that our moderator (i.e., CSR practices) is considered as a group or organizational-level variable, future research should take a multi-level approach.

## 6. Conclusions

The aim of this paper was to investigate the underlying mechanism of the relationship between work overload and turnover intention from the perspective of positive psychology. To empirically test the hypotheses, we used 3-wave time-lagged data from South Korean workers. By performing a moderated mediation model analysis with an SEM technique, we demonstrated that the degree of meaningfulness of work functions as a mediator in the work overload-turnover intention link. Also, CSR activities moderate the work overload-meaningfulness of work link.

Although this paper has some limitations, this study stands to positively contribute to existing literature in work overload by demonstrating an elaborate intermediating process and its contingent factor in the link. By demonstrating the mediating role of meaningfulness of work, this paper explores the underlying mechanism of the link from the perspective of positive psychology. Also, this research reveals that CSR practices, as an organizational level variable, play a buffering role by reducing the negative effects of work overload.

## Figures and Tables

**Table 1:** Descriptive Features of the Sample.

Characteristic	Percent
Gender	
Male	51.7.3%
Female	48.3%
Age (years)	
20–29	14.0%
30–39	34.8%
40–49	34.6%
50–59	16.6%
Education	
Below high school	8.4%
Community college	18.6%
Bachelor’s degree	61.2%
Master’s degree or higher	11.8%
Position	
Staff	23.6%
Assistant manager	21.6%
Manager or deputy general manager	33.5%
Department/general manager or director and above	21.3%
Tenure (years)	
Below 2	20.8%
2–5	25.5%
5–10	20.0%
10–15	14.9%
15–20	10.4%
Above 20	8.4%

**Table 2:** Descriptive statistics.

	1	2	3	4	5	6	7
1. Gender_T2	-						
2. Education	−0.121 *	-					
3. Tenure_T2	−0.253 **	0.051	-				
4. Position_T2	−0.402 **	0.207 **	0.322 **	-			
5. Work Overload_T1	−0.131 *	−0.032	0.077	0.025	-		
6. Turnover Intention_T3	0.139 **	0.002	−0.168 **	−0.102	0.235 **	-	
7. Meaningful of Work_T2	−0.132 *	0.162 **	0.122 *	0.233 **	−0.123 *	−0.404 **	-
8. CSR_T1	−0.151 **	0.107 *	0.196 **	0.148 **	−0.101	−0.262 **	0.352 **

Note: * *p* < 0.05. ** *p* < 0.01.

**Table 3:** Direct, Indirect, and Total Effects of Final Research Model.

Model	Direct Effects	Indirect Effects	Total Effects
Work overload -> Turnover intention	0.221	0.045	0.266

All values are standardized.

## Data Availability

No new data were created or analyzed in this study. Data sharing is not applicable to this article.

## Appendix A. Measures

1. Work overload [59,60] (time point one, gathered from employees)

(a) “I am pressured to work long hours”.
(b) “I have unachievable deadlines”.
(c) “I have to work very fast”.
(d) “I have to work very intensively”.
(e) “I have unrealistic time pressures”.

2. Meaningfulness of Work [22,23] (time point 2, collected from employees)

(a) “The work that I do is meaningful”.
(b) “The work that I do makes the world a better place”.
(c) “My work is one of the most important things in my life”.
(d) “I would choose my current work life again if I had the opportunity”.
(e) “The work that I do is important”.

3. Turnover Intention of Employees [61] (time point 3, collected from employees)

(a) “How likely is it that you will look for a job outside of this organization during the next year?”
(b) “How often do you think about quitting your job at this organization?”
(c) “If it were possible, how much would you like to get a new job?”.

4. Corporate social responsibility [62,63] (time point one, gathered from employees)

4.1. For the environmental dimension,

(a) “Our company participates in activities which aim to protect and improve the quality of the natural environment”.
(b) “Our company implements special programs to minimize its negative impact on the natural environment”.
(c) “Our company targets sustainable growth which considers future generations”.

4.2. For the community dimension,

(a) “Our company contributes to campaigns and projects that promote the well-being of society”.
(b) “Our company emphasizes the importance of its social responsibilities to society”.
(c) “Our company actively participates in voluntarily donations to charities and nongovernmental organizations”.

4.3. For the employee dimension,

(a) “Management at our company is primarily concerned with employees’ needs and wants”.
(b) “Our company policies encourage employees to develop their skills and careers”.
(c) “Our company supports employees’ growth and development”.

4.4. For the customer dimension,

(a) “Our company respects consumer rights beyond legal requirements”.
(b) “Our company provides full and accurate information about its products to its customers”.
(c) “Customer satisfaction is highly important for our company”.
